# Harnessing the potential of African youth for transforming health research in Africa

**DOI:** 10.1186/s12992-024-01039-7

**Published:** 2024-04-25

**Authors:** Yusuff Adebayo Adebisi, Nafisat Dasola Jimoh, Archibong Edem Bassey, Hassan Olayemi Alaka, Mohamed Marah, Chimwemwe Ngoma, Isaac Olushola Ogunkola, Oumnia Bouaddi, Idahor Courage, Radwa Abdalla Abdelatif El-Abasiri, Rime Boutahar, Molly Unoh Ogbodum, Aniekan Michael Ekpenyong, Theogene Uwizeyimana, Oviri Edith Oghenerukevwe, David Bamidele Olawade

**Affiliations:** 1 Research Department, Global Health Focus Africa, Kigali, Rwanda; 2 College of Social Sciences, University of Glasgow, Glasgow, Scotland, United Kingdom; 3 African Young Leaders for Global Health, Abuja, Nigeria; 4 Warwick Medical School, University of Warwick, CV47AL Coventry, United Kingdom; 5 Department of Microbiology, Obafemi Awolowo University, Ile-Ife, Nigeria; 6 Department of Pharmaceutical Sciences, College of Medicine and Allied Health Sciences, Freetown, Sierra Leone; 7 Knowledge-Action-Change, London, UK; 8 Department of Public Health, University of Calabar, Calabar, Nigeria; 9 International School of Public Health, Mohammed VI University of Health, and Sciences (UM6SS), Casablanca, Morocco; 10 Barking, Havering and Redbridge NHS Trust, Romford, UK; 11 Nuffield Department of Population Health, University of Oxford, Oxford, UK; 12 Mohammed V University of Rabat, Rabat, Morocco; 13 Research Department, Global Health Focus Africa, Kigali, Rwanda; 14 Bill and Joyce Cummings Institute of Global Health, University of Global Health Equity, Butaro, Rwanda; 15 Department of Medicine and Surgery, Ladoke Akintola University of Technology, Ogbomosho, Oyo State Nigeria; 16 Department of Allied and Public Health, School of Health, Sport and Bioscience, University of East London, London, UK

**Keywords:** Health research, Youth, Early-career researchers, Global health

## Abstract

Africa faces a significant burden of infectious diseases, including Malaria and HIV/AIDS, along with an increasing prevalence of non-infectious diseases such as diabetes and cancer. This dual health challenge is amplified by socioeconomic difficulties, restricted access to healthcare, and lifestyle changes, thus present unique scientific needs. Effectively addressing these issues requires a skilled scientific workforce adept in comprehensive healthcare strategies. This analysis explores the critical landscape of health research in Africa, emphasizing the unique opportunity presented by the continent’s youthful population, projected to reach almost 1 billion by 2050. The youth’s innovative potential and fresh perspectives offer a chance to overcome development barriers in health research. Nevertheless, challenges such as under-resourced education, limited research training, inadequate mentorship, and funding difficulties persist. This paper urgently calls upon African leaders, international partners, and stakeholders to prioritize health research, mobilize funding, forge strategic partnerships, and empower the youth as essential steps to capitalize on the continent’s dynamic youth for breakthrough health outcomes. Such investments are vital not just for health but for the overall economic, social, and strategic growth of the continent. Through shared responsibility and a united effort, the potential of African youth can be harnessed, leading to transformative research, improved health outcomes, and a prosperous future. This perspective represents the collective voice of passionate young researchers and advocates across Africa, calling for a new era of health research on the continent.

## Introduction

Despite being home to over 17% of the global population, the African continent only contributes a mere 2% in terms of global research output [1]. The continent faces a significant burden of the world’s diseases, particularly communicable diseases such as HIV/AIDS, malaria, and tuberculosis, along with an increasing prevalence of non-communicable diseases such as cancer [2]. The harsh reality encapsulated in these statistics is referred to as the 10/90 gap. This refers to the inequity where 10% of global health research funds are devoted to health problems that account for 90% of the global disease burden - a substantial part of which falls on Africa [3]. Consequently, this gap implies that Africa is heavily reliant on health research conducted in high-income countries that are often not tailored to the specific health challenges and unique socio-cultural contexts found in Africa [4, 5]. African countries, being highly reliant on health interventions based on research conducted in different environments, often encounter difficulties in effectively implementing these strategies due to variations in disease epidemiology and genomics, health system structures, and cultural practices [2, 3]. Addressing this gap requires promoting local innovation and youth engagement, capitalizing on Africa’s future potential.

Considering challenges like the disproportionate research output, the disease burden, the 10/90 funding disparity, reliance on externally sourced research, and implementation hurdles, it is important to acknowledge the tireless efforts of African researchers who, against all odds, continue to make meaningful strides. They navigate these challenges and consistently strive to generate knowledge and solutions tailored to Africa’s context. Yet, these efforts are stifled by the minimal representation of Africa in global health research. This lack of representation inhibits the development of a robust research culture, undermines the capacity-building of African researchers, and stifles innovation targeted at solving the continent’s health challenges. Increasing Africa’s contribution to global health research advances equity and empowers the continent to generate context-specific knowledge and solutions to its health challenges [2, 4, 5]. It ensures that resources are more effectively utilized, interventions are contextually appropriate, and health outcomes are improved.

Investing in and broadening scientific expertise is key in Africa, especially as the continent experiences a demographic shift towards a younger population, notably those aged 15 to 24. With nearly 60% of its population under 25, Africa has the world’s youngest demographic, and it is expected that by 2030, African youth will make up more than 40% of the global youth population. By focusing on education and fostering a passion for scientific inquiry among these young individuals, they can be empowered with the skills necessary to address Africa’s challenges, essential for harnessing the potential of this demographic shift. This commentary represents a demand to the conscience and responsibility of our leaders to prioritize investment in health research. Of particular significance is the perspective of the youth. As the largest demographic in Africa, the youth have an important stake in the decisions made today. They represent the continent’s future leaders, innovators, researchers, and healthcare professionals. However, their potential remains largely untapped due to limited opportunities for conducting research. This perspective is an urgent call for recognition of young people’s capability, ambition, and essential role in driving health research forward on the continent.

## Health research and youth involvement in Africa

**Fig. 1:**
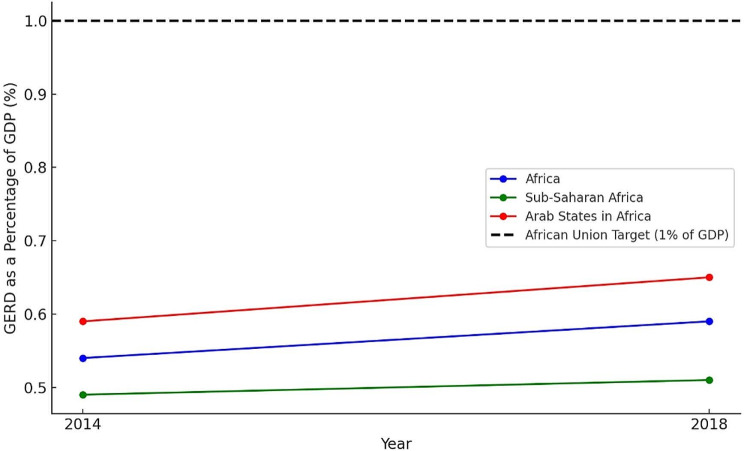
Gross Expenditure on Research and Development (GERD) as a percentage of GDP for Africa, sub-Saharan Africa, and Arab States in Africa from 2014 to 2018. *Data Source* UNESCO Science Report [6]. *Note* The line connecting the data points for 2014 and 2018 is for visualization purposes only and does not imply linearity or any specific trend between these years

To accurately comprehend the current state of health research in Africa, it is vital to evaluate the challenges, acknowledge the advancements, and compare them with global standards. The most formidable obstacle that arises is the insufficient funding, which also extends to a major obstacle to youth involvement in health research. According to the latest UNESCO science report, despite a global rise in spending on science and the number of scientists in the past five years, a trend amplified by the COVID-19 pandemic, no African country is allocating 1% of its Gross Domestic Product towards research and development [5, 6] (See Fig. 1). Inadequate funding for health research in Africa leads to dependency on external donors, skewing research towards global rather than local needs and limiting young researchers’ contributions. This focus may neglect important health areas and leaves young researchers at underfunded institutions with scarce career development opportunities. Such limited investment undermines research quality and impacts, significantly affecting research directions and the support for young African researchers. The global call to decolonize global health research strongly aligns with the imperative to invest in the capacities of African young researchers. This alignment implies a crucial shift: recognizing and rectifying how traditional funding and research paradigms have often marginalized African perspectives, especially those of the youth. Experts have also suggested that the current model of philanthropic aid for health research could perpetuate Africa’s dependence on the Global North and to break this dependency, African stakeholders must develop and implement clear strategies, taking the lead in decisions [4].

The state of health research in Africa can be further illuminated by examining the number of researchers and their density per million inhabitants. Despite the strides made globally, sub-Saharan Africa’s share of global researchers remains small, moving from 0.6% in 2014 to 0.7% in 2018 [5]. However, this still represents a substantial increase in researcher density per million inhabitants for the region - from 102 in 2014 to 124 in 2018. Several African countries showed promising growth and were among the 38 worldwide that had increased their researcher density per million inhabitants by over 15% between 2015 and 2018 [5]. These statistics point to a promising growth in research capacity within the African continent. Yet, for young African researchers, this data also underscores a pressing concern. Despite an increase in researcher numbers, Africa’s youth remain underrepresented in global health research, leading to a skewed understanding of health challenges. More researchers do not necessarily mean a stronger global voice, particularly when young contributions are overlooked. Additionally, disparities in researcher density across countries create unequal opportunities for young researchers, affecting access to resources, training, and mentorship, and contributing to an uneven playing field within the continent.

Another critical obstacle facing health research in Africa is the high emigration rate of skilled researchers, a problem known as brain drain [7–9]. Over 10% of sub-Saharan Africans with graduate degrees emigrate, with numbers significantly higher in the healthcare workforce [9]. This exodus is often driven by factors such as limited local funding and training opportunities, as well as pull factors such as more appealing research environments and working conditions in high-income countries. Retention of professionals with specific technical skills is vital for a thriving health research landscape, making it paramount for African leaders to invest more in its youthful population. Furthermore, the lack of investment in Research & Development (R&D) by African companies, particularly in the health sciences field is another prominent challenge. Globally, pharma companies are leading investors in R&D. However, in Africa, only few companies have R&D units or directors to oversee product creation, knowledge, and technology transfer [10]. Even though Africa is blessed with vast medicinal plants, the research landscape into the health benefits of these plants is still underexplored. This lack of institutional support further hinders the development and growth of health research on the continent. Overall, bolstering the health research ecosystem in Africa requires a comprehensive approach that addresses funding, capacity development, brain drain, and local industry investment [11]. Critiques of capacity development in Africa focus on the disconnect between donor-driven programs and the continent’s specific needs. These efforts, while aimed at empowerment, often fail to consider the complexities of local health systems and cultures, risking dependency rather than fostering sustainable growth.

Africa’s health research is at a critical point, needing to strengthen its global role. Fonn et al. [2] note Africa’s small research output relative to its population, suggesting a need for greater global research participation. Ezeh et al. [1] and the establishment of Consortium for Advanced Research Training in Africa (CARTA) represent steps toward enhancing health research capacity in Africa through a multidisciplinary approach, emphasizing the importance of youth engagement in addressing complex health challenges. Ndejjo et al. [7] bring forth the imperative of positioning Africa’s public health doctoral students as leaders poised to drive societal transformation and development. The youth, being at the helm of innovative solutions and possessing a contemporary understanding of prevailing health issues, are indispensable in the quest to elevate Africa’s health research landscape. Their involvement will not only enriches the research ecosystem but also propels the continent towards homegrown solutions, as underscored by Christoffels [12]. Thus, youth involvement is not a mere accessory but a fundamental requisite to accelerate Africa’s trajectory in global health research and, by extension, its impact on public health outcomes.

Despite these constraints, Africa has demonstrated that it has the talent and ambition to make significant contributions to health research [11]. From pioneering work in managing HIV/AIDS to ground-breaking research in malaria and COVID-19 response, African researchers have made strides in addressing some of the continent’s most pressing health challenges [13–15]. Initiatives like the African Academy of Sciences’ Grand Challenges Africa, Global Health Focus University Excellence, the African Research Excellence Fund, and the Alliance for Accelerating Excellence in Science in Africa showcase the potential of targeted investments in engaging young people and fostering local innovation. Despite signs of growth, the current investment in health research is insufficient to meet the Sustainable Development Goals and Africa’s Agenda 2063.

### Empowering youth: a catalyst for health research and sustainable development in Africa

Recognizing the value of investing in health research is fundamental in prioritizing its development. The potential dividends extend beyond the health sector, promising significant economic, social, and strategic returns. Youth involvement can significantly augment the value of health research by injecting fresh perspectives, innovative ideas, and a dynamic energy into the sector as well as more opportunities for development in Africa.

Health research investment can play a significant role in stimulating economic growth and development. This funding nurtures a broad spectrum of high-skilled jobs that range from laboratory scientists to public health experts, thereby reducing unemployment rates and boosting economic productivity. Moreover, investing in health research can spur technological advancement and innovation, particularly in fields like biotechnology, pharmaceuticals, and healthcare delivery systems [16]. The creation of new treatments, health interventions, and advanced medical devices can result in profitable commercial applications, creating new industries and market opportunities. Such investment can also stimulate an ecosystem of spin-off industries, including data management, logistics, regulatory affairs, and intellectual property management. This ripple effect can result in further job creation and economic development.

From a social perspective, health research is pertinent to achieving better health outcomes and reducing health disparities [17]. Most of the knowledge needed to solve Africa’s health research problems reside in the confines of the continent itself. Balancing local and international research collaboration is key to Africa’s health research development. While local research ensures cultural and contextual relevance, international partnerships bring global expertise and resources. This synergy enhances capacity building, ensuring that local insights are integrated into global health discourse without losing their unique value. Investing in the capacity of young people within the health research sector can foster a locally-driven approach to tackling health disparities, leveraging the inherent knowledge and cultural understanding present within the continent. Through their active involvement and development, youth can contribute to innovative solutions and community engagement strategies that are tailored to Africa’s unique health challenges, thereby enhancing the social impact and relevance of health research in improving health outcomes. Strategic investment in health research holds critical importance for Africa, paving the way for African youth to spearhead efforts in investigating diseases that predominantly afflict the continent, instead of depending on expertise or evidence from other regions. This proactive stance enables African youth to address endemic diseases with heightened precision and efficacy, a necessity given the escalating burden of both infectious and non-infectious diseases. Furthermore, looming global health challenges such as climate change and rapid urbanization are anticipated to usher in unprecedented health issues ranging from heat-related illnesses to pollution-induced respiratory diseases.

The COVID-19 pandemic has accentuated the importance of nurturing a sturdy, home-grown research capacity capable of promptly responding to emerging health crises [18–21]. Without robust health research infrastructure, Africa risks over-reliance on global health initiatives that might not align with its unique needs. Strengthening this infrastructure is key for empowering African youth with tools to understand and address specific health challenges, ensuring they can effectively protect public health in changing conditions. Investing in health research is, therefore, not just a matter of health policy but an imperative for economic development, social equity, and strategic foresight. To harness these benefits, a significant commitment from regional leaders is required.

### The youth perspective and potential

With a youth population projected to reach almost 1 billion by 2050, Africa boasts the youngest demographic in the world [22]. This emerging powerhouse of youthful energy and innovative potential presents an extraordinary opportunity for the continent to leapfrog development barriers. Among the manifold areas for youth engagement, health research stands as a pivotal field where young Africans can significantly contribute towards reshaping their communities and countries [11]. African youth offer unique insights into health challenges based on their lived experiences, understanding the broader impact on economy, society, and quality of life. Developing a pipeline of young researchers will position Africa not just as a research subject but as a key player in the global health arena.

Despite the clear advantages that youth involvement offers, they face several barriers that often stifle their potential. These challenges range from under-resourced educational institutions and limited opportunities for advanced research training, to inadequate mentorship– often due to over-worked or insufficiently qualified senior staff - and the perpetual struggle for research funding [23–25]. Consequently, many young Africans who are interested in health research are left unsupported and underutilized. Under-resourced institutions and the lack of quality education are critical barriers. Many African universities and research institutions lack the capacity to provide high-quality health research training [26]. Limited access to up-to-date resources, inadequate research infrastructure, and the dearth of experienced research mentors further compound these challenges. This situation stifles the intellectual growth of young researchers and hinders their ability to make meaningful contributions to the field of health research.

In addition to education, funding is a fundamental challenge. Despite the vast potential that young African researchers offer, securing funding for their research projects is often difficult [24]. Young researchers typically lack the track record and experience that grant review committees look for. As a result, they often struggle to compete with established researchers for limited funding resources. This lack of financial support not only stifles their current research activities but also threatens to discourage them from pursuing a career in health research altogether. Addressing these challenges requires comprehensive, systemic solutions. Improving the quality of education and research training can create a conducive environment for young researchers to grow and thrive [11]. This might entail investing in research infrastructure, updating curriculum to meet international standards, providing access to digital resources, and fostering partnerships with international institutions for research collaboration and faculty development. Additionally, creating more dedicated funding streams and grants for young researchers, such as the Royal Society of Tropical Medicine and Hygiene Early-Career Small Grants, can enable them to kickstart their research careers. Mentorship programs that connect young researchers with established scientists can further support their professional growth. Encouragingly, several initiatives across Africa are paving the way for youth engagement in health research. Programs such as the African Research Leaders programme, Global Health Emerging Leaders Programme, Global Health Mentorship, and the Developing Excellence in Leadership, Training, and Science Africa programme among others, are fostering the next generation of African health researchers. These efforts, however, need to be scaled up and complemented by national policies that prioritize youth involvement in health research.

## Call to action

The urgent necessity for progress in health research within Africa, interlinked with the untapped vigor of the continent’s youthful demographic, highlights an immediate call to action. This analysis beckons African leaders, international allies, and youthful stakeholders to envisage and foster a future where Africa transitions from being a mere participant in global health research to a formidable driver. The realization of this vision demands the adoption of certain key strategies and commitments.

Initially, this call champions African leaders to prioritize health research. It is essential to enact and enforce policies that allocate at least 3% of National Health Expenditures to health research—a benchmark inspired by our recommendations, adjusted to meet the escalating health research demands on the continent. Establishing dedicated national research funds and innovative financing channels is key to ensure a sustainable funding stream for health research and an indispensable opportunity to contribute to the capacity building of African youth. Moreover, cultivating strategic partnerships across sectors—encompassing governments, academic institutions, private entities, and civil society—is essential. For instance, a pragmatic step could be the initiation of an Africa-wide biobank through collaborative endeavours among African nations, akin to the successful model of the UK Biobank. This initiative could catalyse knowledge exchange and capacity building among African young researchers, propelling significant health outcome advancements and elevating Africa’s stature in the global health research domain [27]. Aligned with the Africa 2063 agenda, nurturing critical research skills from early stages of education through tertiary levels is pertinent [11]. Investment in undergraduate research capacity is pivotal to instill a culture of inquiry and innovation. Governments, academic institutions, and stakeholders should also foster postgraduate research opportunities targeting global public health issues. Initiatives like the African Research Excellence Fund [28], which supports early-career researchers, are commendable and should be emulated and scaled up.

A shift in perspective is key to champion the advancement of health research in Africa, delineating a clear and active role for the young people as catalysts of the envisaged changes, rather than mere beneficiaries. Youth-led initiatives, akin to the ‘Young Professionals Chronic Disease Network’, have showcased the capability of young minds in driving global health agendas. This call to action emphasizes the proactive engagement of young individuals in research activities, policy advocacy, and community outreach. As advocates of youth involvement in health research, we propose transformative strategies that could revolutionize the youth’s contribution. These are:

1. **Youth-Led Health Research Innovation Hubs**: Advocate for the establishment of Youth-Led Health Research Innovation Hubs across African nations. These hubs could serve as incubation centres for young researchers to develop and pilot innovative health research methodologies and technologies, fostering a culture of innovation and problem-solving tailored to local health challenges.
2. **Community-Engaged Research Initiatives**: Promote and support youth-led, community-engaged research initiatives that focus on grassroots problem-solving. By working closely with local communities in identifying and addressing health issues, research becomes more relevant, culturally sensitive, and impactful at the local level.
3. **Digital Health Research Platforms**: Propose the creation of digital home-grown platforms for African young researchers to collaborate, access resources, and share their findings globally, democratizing health research and making it more accessible and inclusive.
4. **Policy Advocacy and Youth Representation**: Stress the importance of youth representation in health research policy-making bodies at both national and international levels. With a seat at the table, young researchers can advocate for policies that foster innovation, inclusivity, and equity in health research.
5. **Cross-disciplinary and Inter-generational Mentorship Programs**: Support and strengthen mentorship programs that promote cross-disciplinary and inter-generational collaborations, providing a platform for knowledge exchange and a holistic approach to health research.
6. **Crowdsourced Research Funding**: Introduce the concept of crowdsourced funding for youth-led research projects as an alternative funding mechanism, allowing the public to directly support innovative research projects.
7. **Competitive Innovation Challenges**: Organize competitive innovation challenges to stimulate creative thinking, collaboration, and the practical application of research skills among young researchers.

Globalization presents both challenges and opportunities for African youth in health research. While it can lead to marginalization by prioritizing international agendas, it also opens doors for cross-border collaborations and diaspora partnerships, enriching the research landscape with diverse insights and resources [29–31]. As this call-to-action advocates for increased youth involvement in health research, a spotlight on equity is indispensable to ensure balanced representation inclusive of marginalized communities and individuals. This approach transcends bridging health research gaps; it aims at addressing disparities in contributions among different youth groups, thereby promoting a more equitable research landscape [32]. Envisioning the future, this call foresees Africa steering global health research, with African researchers, buoyed by youthful zest, innovation, and resilience, leading transformative research endeavours. They will operate within well-resourced institutions, engage in productive global collaborations, and significantly influence policy and intervention strategies improving health outcomes. The promise of a thriving health research milieu in Africa, navigated by the enthusiasm and potential of the youth, heralds a healthier future for the continent. All stakeholders are beckoned to join in this mission, acknowledging the significance of investing in health research and youth empowerment.

## Conclusion

As this perspective concludes, the urgency and importance of the message are reiterated. Health research in Africa is significantly underfunded and underprioritized, yet its potential to transform health outcomes, drive economic growth, and foster social development is immense. Moreover, the pivotal role of African youth in driving this transformation is emphasized. The urgency of this article stems from the stark reality of the continent’s health challenges and the need for home-grown solutions. The call for increased investment, a whole policy framework, and international collaboration in health research is an imperative. Ignoring this call risks perpetuating health disparities, impeding economic progress, and leaving the continent ill-prepared for future and current health crises. As young researchers, they are not only the beneficiaries of these proposed changes but also the drivers. They are ready to take up the mantle of leadership, equipped with ambition, innovative spirit, and deep understanding of the continent’s health needs. Their voices and perspectives are essential in shaping the future of health research in Africa.

## Data Availability

Not Applicable.

## Abbreviations

HIV/AIDS: Human Immunodeficiency Virus/ Acquired Immunodeficiency Syndrome
UNESCO: United Nations Educational, Scientific and Cultural Organization
COVID-19: Coronavirus Disease
CARTA: Consortium for Advanced Research Training in Africa
